# Investigating the feasibility of stratified breast cancer screening using a masking risk predictor

**DOI:** 10.1186/s13058-019-1179-z

**Published:** 2019-08-09

**Authors:** Olivier Alonzo-Proulx, James G. Mainprize, Jennifer A. Harvey, Martin J. Yaffe

**Affiliations:** 1 Physical Sciences, Sunnybrook Research Institute, 2075 Bayview Avenue, Toronto, Ontario M4N 3M5 Canada; 2 Department of Radiology and Medical Imaging, University of Virginia Health System, Charlottesville, VA USA; 3 Department of Medical Biophysics, University of Toronto, 2075 Bayview Avenue, Toronto, Ontario M4N 3M5 Canada

**Keywords:** Breast density, Masking, Stratified screening, Detectability, Interval cancers

## Abstract

**Background:**

Women with dense breasts face a double risk for breast cancer; they are at a higher risk for development of breast cancer than those with less dense breasts, and there is a greater chance that mammography will miss detection of a cancer in dense breasts due to the masking effect of surrounding fibroglandular tissue. These women may be candidates for supplemental screening. In this study, a masking risk model that was previously developed is tested on a cohort of cancer-free women to assess potential efficiency of stratification.

**Methods:**

Three masking risk models based on (1) BI-RADS density, (2) volumetric breast density (VBD), and (3) a combination of VBD and detectability were applied to stratify the mammograms of 1897 cancer-free women. The fraction of cancer-free women whose mammograms were deemed by the algorithm to be masked and who would be considered for supplemental imaging was computed as was the corresponding fraction in a screened population of interval (masked) cancers that would be potentially detected by supplemental imaging.

**Results:**

Of the models tested, the combined VBD/detectability model offered the highest efficiency for stratification to supplemental imaging. It predicted that 725 supplemental screens would be performed per interval cancer potentially detected, at an operating point that allowed detection of 64% of the interval cancers. In comparison, stratification based on the upper two BI-RADS density categories required 1117 supplemental screenings per interval cancer detected to capture 64% of interval cancers.

**Conclusion:**

The combined VBD/detectability models perform better than BI-RADS and offer a continuum of operating points, suggesting that this model may be effective in guiding a stratified screening environment.

## Background

High mammographic density is associated with increased risk of developing breast cancer [1–5] and also reduces the diagnostic accuracy of mammography due to masking [6–8]. Compared to women with fatty breasts, women with dense breasts are at least 3.5 times more likely to have an interval cancer, one diagnosed less than 1 year after a negative screening mammogram [9]. Thirty-six states in the USA have now enacted Density Notification Laws, most requiring that women be informed when they have dense breasts. Many include language stating that high breast density may affect the diagnostic value of their mammogram and that supplemental screening exams could be considered. A method of identifying those women for whom mammography will not provide adequate detection of breast cancer could provide a means for guiding those women toward an alternative or supplementary method that would yield better performance.

A stratification method must be both effective (identifying women where the risk of missed detection of breast cancer is high due to masking) and efficient in referring as few women as possible who do not have breast cancer for supplementary screening. Breast density is typically assessed using the Breast Imaging Reporting and Data System (BI-RADS) scale [10, 11], in which mammograms are subjectively categorized in four groups by a radiologist. Women in the two upper categories, i.e., with heterogeneously dense or extremely dense mammograms, are considered to have dense breasts, which corresponds to approximately 40% and 8% of mammograms, respectively [12]. Thus, approximately half of screened women are potentially eligible for supplemental screening. Kerlikowske et al. [12] have argued that due to this high prevalence, rather than stratifying on BI-RADS density category alone, efforts should be focused to women at most risk for an interval cancer.

There are two types of risk associated with elevated density, the underlying breast cancer risk and the risk of masking. Here, we attempt to isolate the masking risk and create a model that can be used to identify women for whom mammography screening will likely be compromised due to masking. The two types of risk can then be evaluated and used separately in the optimization of strategies for screening.

We have reported previously on the development of a “masking risk” model [13], derived from biometric and image-based parameters that can discriminate between the mammograms associated with screen-detected (SD) cancers and those associated with non-screen-detected (NSD) cancers, i.e., those which were found by other means less than 13 months after a negative screening examination. Most women screened do not have breast cancer, and the feasibility of stratified screening would depend on identifying as few of these women as possible for supplemental screening. In this investigation, the model is applied to mammograms from cancer-free women to evaluate the effect of decision thresholds on the efficiency of stratifying women at greatest risk for interval cancer to supplemental screening.

## Methods

### Study population

The mammograms used in this study came from an earlier study where breast density was incorporated in a risk stratification model for breast cancer [14]. This study had institutional review board approval. All patients underwent informed consent for participation in the original study. The need for additional consent for this study was waived, as only existing de-identified data were used in this analysis. Both studies were compliant with the Health Insurance Portability and Accountability Act.

In the original study [14], all women diagnosed with cancer at a single US institution between 2003 and 2013 and with a digital contralateral mammogram at the time of diagnosis were eligible as cases. These were matched to cancer-free controls, defined here as having had two consecutive negative screening mammograms (i.e., both the index mammogram and the next screening mammogram were negative). Women in the study were participant in an annual screening program. Women who agreed to participate were asked to complete a questionnaire which included age and body mass index (BMI) information. BI-RADS density category (fourth edition) was obtained from the mammography report. Volumetric breast measures including total breast volume and percent breast density by volume (VBD) were measured using automated commercial software (Volpara 1.5.0, Volpara Solutions, Wellington, NZ).

The calculations were performed on the index unprocessed, i.e., “DICOM For Processing” mammograms, which included at least one standard view (craniocaudal or mediolateral oblique). Tiled views of large breasts were excluded. Arbitrarily, the measurements were performed on the left breast by default, except when the left views were not available. When multiple views were obtained for one projection, the values were averaged. Initially, there were images from 2047 cancer-free women: 4 were excluded because of tiled views; 31 and 11 were excluded due to failure of the Volpara calculation or our detectability algorithm (described below), respectively; 104 were excluded due to missing BI-RADS data, resulting in the images of 1897 women available for analysis.

### Masking risk algorithm

The development of the masking risk algorithm was previously described in detail [13], but is summarized here. (1) In a case-case analysis of 70 SD cases and 44 NSD cases, the NSD or SD status was used as a surrogate indicator that masking has occurred or not; (2) BI-RADS density category and Volpara VBD and breast volume were estimated on the mammograms; (3) “Maps” were calculated for each mammogram, showing the spatial distribution of VBD (in this case, calculated using an in-house algorithm) and local detectability as described below; (4) Statistical and texture metrics were derived from the VBD and detectability maps; (5) Stepwise multivariate logistic regressions were performed to determine which of the metrics yielded the best classification performance between NSD and SD cases. Cases in the categories were not matched for age; however, age was a covariate in the multivariate modeling. Each regression produced a predictor of masking risk: a variable-threshold classifier that rates the likelihood of a mammography exam as being masked (i.e., being an NSD case) or non-masked (an SD case).

In the modeling [13], the assumption was made that the NSD cancer cases correspond to interval cancers in their broad definition, which includes masked (i.e., missed or false negative) cancers and “new” cancers that are found between regular annual screens. To allow for the variability of the actual interval at which women present for their screening examinations, the window for inclusion of interval cancers was extended to 13 months. Because these cancers were found by other means less than 13 months after a negative mammogram and the mean sojourn time of breast cancer is 2.0 years (95th percentile = 150 days) [15, 16], it is estimated that nearly all of these are in the false negative category.

To predict how many women without breast cancer would be recommended for supplementary screening with each of the masking prediction models, it is necessary to run the algorithms on sets of normal cases. The three models: “BI-RADS Density,” “Adjusted Volpara,” and “DETECT+,” under consideration for use for stratification are summarized in Table 1. The ultimate covariates selected for each model are listed in the first column. DETECT+ is an in-house algorithm that was found in [13] to be the best performing model describing masking probability. Note that all of the models incorporate some measure of breast density. DETECT+ employs a specially designed volumetric density algorithm described by Mainprize et al. [17].

**Table 1:** Details of the predictive models for the 1-year interval cancers showing the predictors from each model. 95% confidence intervals are presented in brackets. The odds ratio corresponds to the relative odds between the first and last quartile for the continuous predictors which are used as covariates in the multivariate models. For BI-RADS density, the odds ratio corresponds to the relative odds between the specified BI-RADS category and the reference category, BI-RADS 1. AUC refers to the area under the receiver operating characteristic curve

Selected predictors	Odds ratio		AUC	
BI-RADS density				
BI-RADS 3:1	9.78	[2.16–44.38]	0.67	[0.57–0.76]
BI-RADS 4:1	13.33	[2.37–75.15]		
BI-RADS 2:1	6.53	[1.44–29.56]		
Adjusted Volpara				
Age at exam	0.49	[0.30–0.80]	0.74	[0.62–0.83]
Breast volume (Volpara)	0.64	[0.39–1.05]		
VBD (Volpara)	1.40	[0.91–2.17]		
DETECT+				
Detectability Std Dev	0.47	[0.24–0.89]	0.79	[0.69–0.87]
Density GLCM correlation	1.85	[0.98–3.49]		
Age at exam	0.67	[0.40–1.12]		

### Simulation of a stratified screening program

Our approach to stratification is to try to maximize the number of women for whom there is the potential for cancer masking who will be identified for supplemental screening while minimizing the number whose cancers (if present) are expected to be detectable on mammography. These two competing factors are computed to determine the efficiency of a simulated stratified screening environment.

The masking risk is computed on each of the images using the models described above. Each model is tested over a range of candidate thresholds of masking risk. The threshold (or operating point) distinguishes between images that are deemed to be “masked” or “non-masked.” Women identified as “masked” are the ones who would be considered as potential candidates for supplemental imaging. For those women, two fractions are determined: (1) The recruitment fraction (*RF*), which is the fraction of cancer-free women whose mammograms would be rated above the masking threshold, and (2) the capture fraction (CF), the fraction of women from NSD cases whose mammograms are above the same masking threshold. We examine the relationship between *RF* and *CF* at different settings of the threshold. In this analysis, the cancer-free women are used as a proxy for a screening population.

Given an underlying NSD cancer rate (assumed to be equivalent to the interval cancer rate or ICR) in a population of *N* individuals, the maximum number of NSD cancers for potential detection by supplemental imaging is given by *N* × ICR × *CF*. The corresponding number of women considered for supplemental imaging is *N* × *RF*. The number of women considered for supplemental imaging per interval cancer potentially detected is thus *RF*/(ICR × *CF*). This ratio represents the “cost” of the supplemental screening program, in that as more women receive the additional screen (with the benefit of detecting missed cancers), more inconvenience to women and financial costs are imposed on the health care system. The most efficient (or lowest cost) supplemental screening program will have the lowest *RF*:*CF* ratio. See the Appendix for a detailed description of the calculation of masking risk thresholds, *CF* and *RF* for the models discussed here. To evaluate performance, the C-statistic or discrimination accuracy of the masking risk models was computed. This is the probability that a model will score a randomly selected NSD exam at a higher masking risk than a randomly selected cancer-free exam.

For simulating a screening program, an ICR of 0.60 per 1000 screens was estimated using data from the Breast Cancer Surveillance Consortium (BCSC) as reported by Kerlikowske et al. [12]. Here, *N* was set to 100,000. Error estimates on the values of the cost function were estimated by bootstrapping, for 1000 bootstrap replicas, using the “*bootci*” function in Matlab 2016b (Mathworks Inc. Natick, MA).

## Results

Table 2 shows descriptive statistics (age, BMI, BI-RADS density category, and mammography vendor) for the NSD cases and cancer-free women. Women with interval cancers were generally younger, with lower BMI, higher BI-RADS density, and imaged on GE systems compared to the cancer-free women. The *p* value of the difference between the two groups was computed using a two-sample *t* test or chi-square test for the continuous or categorical data, respectively.

**Table 2:** Descriptive statistics of age, BMI, BI-RADS density and mammography vendor for the non-screen detected and cancer-free women

	Non-screen detected		Cancer-free		*p* value
	*N* = 44		*N* = 1897		
	Count	%	Count	%	
Age at mammography					0.036
< 40	3	6.8	41	2.2	
41–50	13	29.5	370	19.5	
51–60	13	29.5	673	35.5	
61–70	8	18.2	563	29.7	
71–80	6	13.6	217	11.4	
> 80	1	2.3	33	1.7	
BMI					0.149
< 22.8	15	34.1	509	26.8	
22.8–26.4	14	31.8	562	29.6	
26.4–30.6	10	22.7	439	23.1	
> 30.6	5	11.4	387	20.4	
BI-RADS density category					0.010
1	1	2.3	375	19.8	
2	15	34.1	713	37.6	
3	22	50.0	632	33.3	
4	6	13.6	177	9.3	
Mammography vendor					0.011
Hologic	5	11.4	549	28.9	
GE	39	88.6	1348	71.1	

Table 3 shows *CF* vs. *RF* for the three stratification models.

**Table 3:**
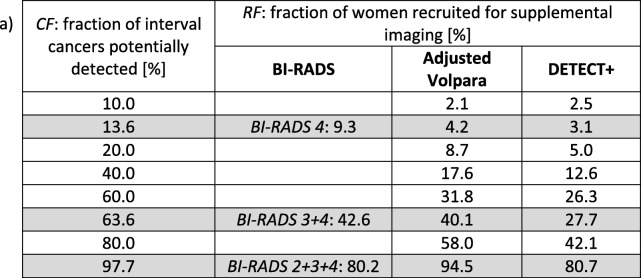
Select operating points of the capture fraction of interval cancers potentially detected (*CF*) vs. the recruitment fraction for supplemental imaging (*RF*) for the three stratification models in a simulated annual screening program. The rows or operating points correspond to specific thresholds for masking risk. The results were linearly interpolated when necessary. Shaded rows align with the three BI-RADS thresholds (i.e., women in density category 4 only; 3 and 4; 2, 3, and 4)

**Fig. 1:**
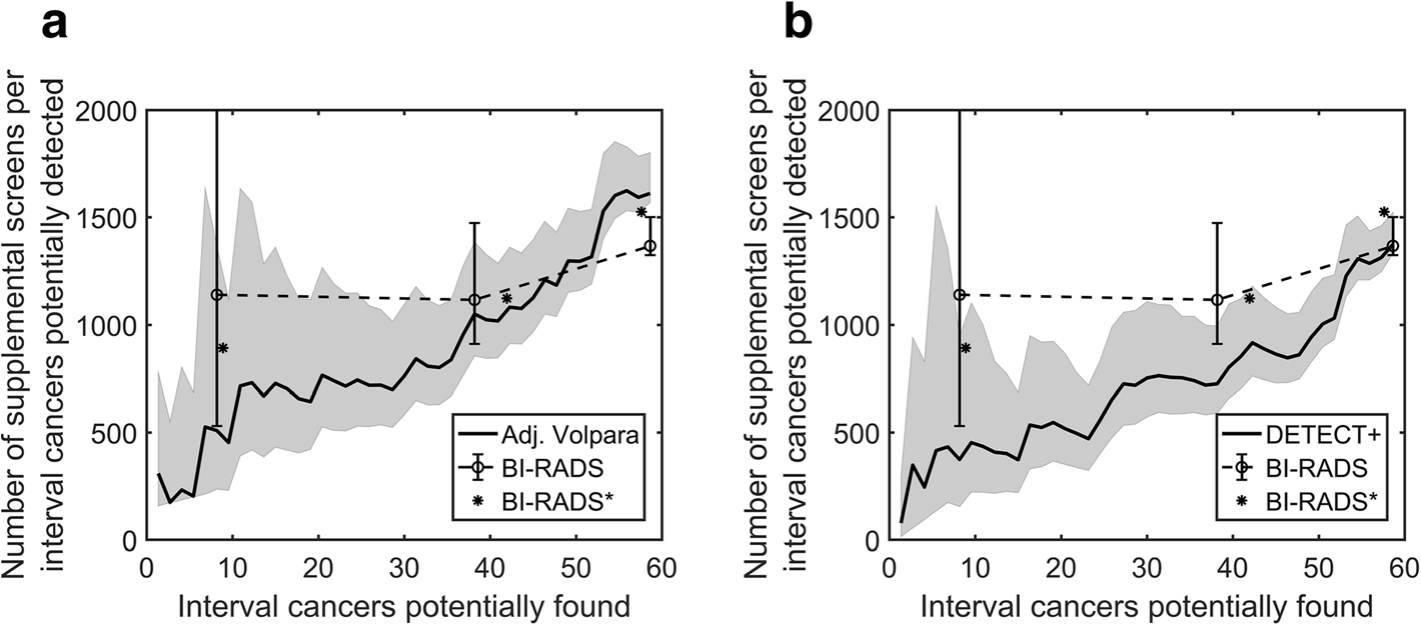
Plot of the cost function (number of supplemental screens per interval cancer potentially detected) vs. the number of interval cancers for potential detection. Left panel: the Adjusted Volpara model; right panel: the DETECT+ model. Data points denoted with an asterisk, obtained from ref. [12] and rescaled, represent the cost function of supplemental screening using BI-RADS density from a large dataset. Shaded region or error bars correspond to the 95% confidence interval

Table 4 shows the corresponding number of interval cancers potentially detected vs. the cost function, expressed as the number of women recommended for supplemental screening per interval cancer potentially detected, assuming a prevalence of 60 interval cancers in 100,000 women screened [12]. This shows that the DETECT+ model generally requires the fewest supplemental exams per interval cancer detected. For example, with a threshold set to identify 38 interval cancers (*CF* = 64%), supplemental screening would be performed for *RF* = 43%, 40%, and 28% of screening participants when supplemental screening is triggered on the basis of BI-RADS 3 or 4 density category, Adjusted Volpara, or DETECT+, respectively. This corresponds to 1117, 1051, and 725 supplemental screens per interval cancer detected. Figure 1 graphically shows the cost function versus the number of interval cancers potentially found for the three stratification models. Stratification data for BI-RADS density extrapolated from Kerlikowske et al. [12] are also shown for comparison. The C-statistic and 95% confidence interval for the BI-RADS, Adjusted Volpara, and DETECT+ models were 0.63 [0.56–0.69], 0.66 [0.56–0.73], and 0.72 [0.65–0.79] respectively.

**Table 4:**
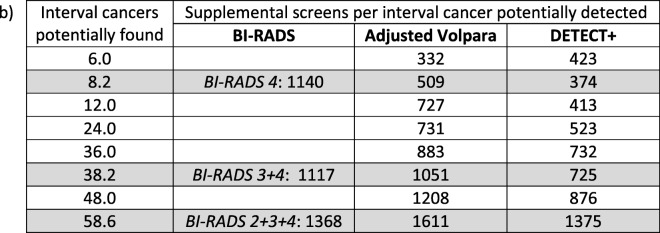
For a cohort of 100,000 screening participants with 60 interval cancers, the cost function (supplemental screens per interval cancer detected) versus the number of interval breast cancers potentially detected at the same operating points as in Table 3). The rows or operating points correspond to specific thresholds for masking risk. The results were linearly interpolated when necessary. Shaded rows align with the three BI-RADS thresholds (i.e., women in density category 4 only; 3 and 4; 2, 3, and 4)

**Fig. 2:**
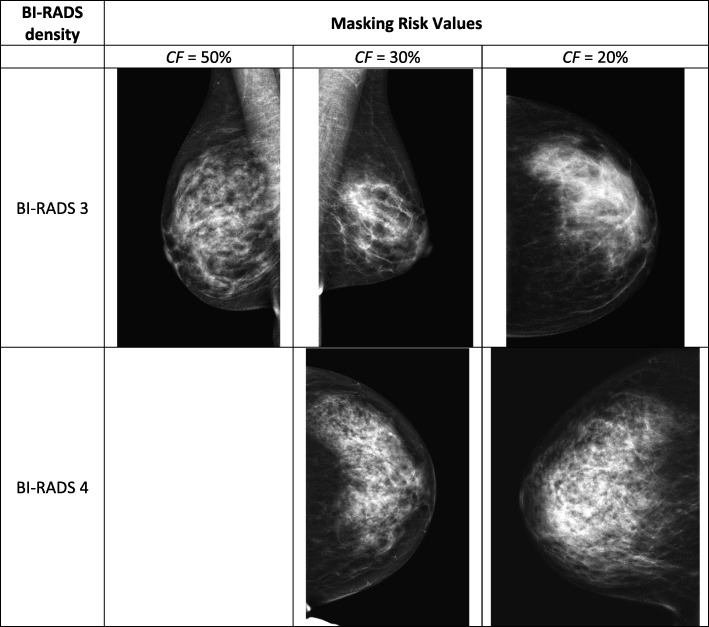
Example mammograms from the interval cancer cohort at different BI-RADS and masking risk values. Images shown are in the “For Presentation” format

Figure 2 shows the contralateral mammograms of women with interval cancers with mammograms rated as BI-RADS 3 (top) and BI-RADS 4 (bottom). In each row, the calculated masking risk increases from left to right. If set as thresholds, they would correspond to *CF* values of approximately 50%, 30%, and 20% respectively for the DETECT+ model.

Table 5 shows data from this work and from two studies by Kerlikowske et al. [12, 18]. It is seen that the three populations have similar distributions in the high BI-RADS density categories for all women or cancer-free women, while Kerlikowske et al. [18] shows a higher proportion of interval cancer cases in the BI-RADS 4 category than the other two studies.

**Table 5:** Comparison of the distribution in the dense BI-RADS categories and the corresponding costs (in supplemental screens per interval cancer detected) for BI-RADS stratification of this work and of other studies

	Kerlikowske et al. [12]			Kerlikowske et al. [18]			This work		
	Percent		Cost	Percent		Cost	Percent		Cost
	Interval cancer	All women		Interval cancer	Cancer-free		Interval cancer	Cancer-free	
BI-RADS 4	14.8	8.0	892	27.9	8.5	508	13.6	9.3	1140
BI-RADS 3	55.4	39.4		45.6	33.3		50.0	33.3	
BI-RADS 3 + 4	70.2	47.4	1124	73.5	41.8	948	63.6	42.6	1117

## Discussion

The DETECT+ model offers the lowest cost over a wide range of capture fraction (CF = 14 to 98%) of interval cancers. The cost for the Adjusted Volpara model is lower only at the relatively low capture fraction of 10% (i.e., 6 of 60 interval cancers potentially detected). For example, the DETECT+ model requires 12,588 supplemental screens (*RF* = 12.6%) to potentially detect 24 interval cancers (*CF* = 40%), representing a costs of 523 supplemental screens per interval cancer. When inverted, this costs represent 1.9 interval cancers potentially detected per 1000 supplemental screens. The Adjusted Volpara model requires, at the same operating point, 39% *additional* supplemental screens to potentially detect the same amount of interval cancers in 100,000 women. The discriminatory accuracy (C-statistic) between interval cancer cases and cancer-free women of the DETECT+ model was the largest at 0.72 [0.65–0.79]. Based on confidence intervals, the DETECT+ model was statistically better than the BI-RADS model, and just short of a statistical difference to the Adjusted Volpara model.

The BI-RADS-based model was considered, primarily because this system is widely used by breast radiologists; however, its major limitations are that it has higher costs and has only three operating points. For example, if the threshold for suggesting supplemental screening was for women with extremely dense breasts (BI-RADS 4) only, 9331 supplemental screens (*RF* = 9.3%) would be required, with 8 of the interval cancers potentially detected (*CF* = 13.6%). This corresponds to a cost of 1140 supplemental screens per interval cancer detected. Interestingly, at the same *CF*, the DETECT+ model would label only approximately 18% of the BI-RADS 4 examinations as masked, yet has a cost of only 374 supplemental screens per interval cancer (*RF* = 3.1%).

This implies that high masking does not necessarily occur in all BI-RADS 4 women, but at the same time high masking can occur in images with lower BI-RADS scores. Although there is a correlation between BI-RADS density and masking, there are additional subtleties that can disrupt this correlation and are revealed through the DETECT+ metric where not only the area of the breast occupied by dense tissue, but also the intensity and texture of tissue attenuation are considered. It is also worth emphasizing that in addition, there can be considerable intra- and inter-observer variability in assigning BI-RADS density categories and that the results may shift systematically if the different definitions of the BI-RADS fifth edition were used.

While the sample population used in this analysis is small, which leads to large uncertainties in the calculated rates and cost functions, it is proportioned similarly in BI-RADS density compared to larger studies [12, 18]. It is noted that small differences in those proportions can have a large impact in the cost function, due to the small-valued interval cancer rate that appears in the denominator.

Holland et al. [19] have performed a similar analysis using a masking model (*DTMM*) that is based on Volpara VBD as well as lesion size and location. The performance of the DETECT+ metric in a supplemental screening environment is similar but marginally better than their results. They also present results using Volpara VBD alone, which outperforms the Adjusted Volpara model shown in this analysis. This finding may be due to the fact that the models of Holland et al. [19] were created from direct comparisons between interval cancer cases and cancer-free controls, and thus may show a compound effect of masking risk and the underlying breast cancer risk, whereas our models were optimized to discriminate between interval and screen-detected cancer cases.

Kerlikowske et al. [18] have also performed a similar analysis, computing the discriminatory accuracy (C-statistic) between interval cancer cases and normal cancer-free controls of BI-RADS and automated BI-RADS (using Volpara VBD), reporting respective accuracies of 0.72 and 0.70. The models were adjusted for multiple breast cancer risk factors. As in the work of Holland et al. [19], the models likely show a compound effect of both masking and breast cancer risks.

By combining BI-RADS density and BCSC 5-year risk, Kerlikowske et al. [12] report a cost of 694 supplemental screens per interval cancer potentially found. After rescaling for a total population of 100,000 women, this corresponds to *CF* = 27.9% and *RF* = 11.5%. At that *CF* value, the DETECT+ model is about 23% more efficient, with a cost of 532 supplemental screens per interval cancer (*RF* = 8.9%).

We believe that it is a strength of the design of the DETECT+ model that it predicts masking risk only, i.e., when mammography is likely to be diminished in accuracy. It may then be used in conjunction with separate established breast cancer risk models to guide breast cancer screening stratification. For example, women with low masking risk would benefit from mammography screening, at possibly different intervals depending on their underlying breast cancer risk. Conversely, women with high masking risk would benefit from more sensitive screening modalities, also at possibly different intervals depending on their breast cancer risk.

An imbalance between NSD and cancer-free groups according to mammography vendor was observed with proportionally more NSD cases imaged with GE systems. A subset analysis performed using the GE data only showed the C-statistic of the DETECT+ model to be unchanged at 0.72 [0.64–0.79]; however, there were a relatively small number of Hologic NSD cases in the set. In ongoing work with a broader data set, we will determine if a system-dependent covariate will improve the model. It is noted that the GE system was introduced to the clinic earlier when experience with digital mammography was quite limited and the majority of the NSD cases come from this time period.

This study has several limitations. In the dataset, there were a small number of cancers with extremely dense BI-RADS category, very high VBD or very low detectability, resulting in the fairly wide confidence intervals seen for low numbers of interval cancers detected. These women, who have the highest masking risk, would likely be offered supplementary screening according to any of the models used.

These models are currently being tested in a larger population for further validation and to predict the costs of supplemental screening. The simplifying assumption was also made that all interval cancers would be detected by supplemental screening tests. While supplemental screening will increase cancer detection [20], some cancers will also be missed by those tests, and some rapidly growing cancers will always evade detection by screening.

## Conclusions

The masking risk estimator presented in this investigations shows good potential for guiding stratification of breast cancer screening. This estimator is more efficient than using BI-RADS density and provides a continuous scale, allowing for optimizing the balance between the number of women receiving supplemental screening versus the number of interval cancers potentially detected.

## Figures and Tables

**Table 7:**
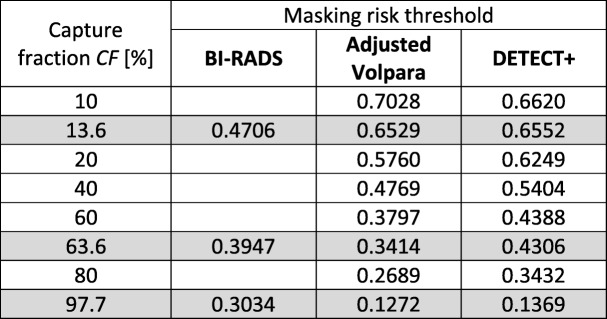
Masking risk thresholds of the models shown in Tables 3 and 4. Shaded rows align with the three BI-RADS thresholds (i.e., women in density category 4 only; 3 and 4; 2, 3, and 4)

## Data Availability

The datasets used and/or analyzed during the current study are available from the corresponding author on reasonable request.

## Appendix

Included here are supplementary data and details of calculation procedures for the three models discussed in the main article, specifically for determining the thresholds for masking risk and for estimating *CF* and *RF* for those thresholds. In our previous work (ref. [13]), logistic regression by a generalized linear model was used to fit binary data (masked cases and non-masked controls), such that the logarithm of the odds of being masked versus non-masked is:

$$ \log \left(\frac{p_i}{1-{p}_i}\right)={\beta}_0+{\beta}_1{x}_{i1}+{\beta}_2{x}_{i2}+\dots, $$

where *p*_*i*_ is the probability that the *i*th subject is a case (i.e., masked), *x*_*ij*_ its corresponding *j*th predictor (e.g., age, BI-RADS density, detectability), *β*_*j*_ the fitted coefficients for each predictor, and *β*_0_ the intercept. Table 6 in the Appendix shows the parameters for the DETECT+ logistic regression model. The parameters for the other models can be found in ref. [13].

**Table 6:** List of the regression coefficients for the DETECT+ logistic model

Model and predictor	Coefficient (*β*_*j*_)
Intercept	− 1.6350
Detectability Std Dev	− 0.0526
Density GLCM correlation	3.8189
Age at exam	− 0.0251

The predictors listed in the table above were computed on cancer-free and masked cancer subjects. For example, cancer-free subject no. 100 had values of 4.38, 0.883, and 50.86 for Detectability Std Dev, density GLCM correlation, and Age at exam, respectively, which leads to a masking risk or probability *p*_100_ = 0.557. Any value of the probability is a potential masking risk threshold *p*_*t*_, allowing the determination of the capture and recruitment fractions *CF* and *RF* at that threshold. For example, at *p*_*t*_ = 0.431, there are 28 (of 44) and 525 (of 1897) subjects with masked cancer and who are cancer-free with *p* > *p*_*t*_, respectively, leading to *CF* = 63.64% and *RF* = 27.68%. Given a screening population of 100,000 individuals and an interval cancer rate of 0.6 per 1000, this corresponds to 27,675 supplemental screens and 38.2 interval cancers potentially detected, for a cost of 724.8 supplemental screens per interval cancer. This masking risk threshold value (or operating point) corresponds to row 6 in each of Tables 3 and 4. The thresholds were obtained by interpolation, increasing the threshold value until a desired CF was obtained. Table 7 in the Appendix lists the thresholds for the three masking models at the operating points shown in Tables 3 and 4.

## Abbreviations

AUC: Area under the receiver-operator characteristics curve
BCSC: Breast Cancer Surveillance Consortium
BI-RADS: Breast Imaging Reporting and Data System
BMI: Body mass index
CF: Capture fraction
ICR: Interval cancer rate
NSD: Non-screen-detected
RF: Recruitment fraction
SD: Screen-detected
VBD: Volumetric breast density
